# Long-Term Access to Sit-Stand Workstations in a Large Office Population: User Profiles Reveal Differences in Sitting Time and Perceptions

**DOI:** 10.3390/ijerph15092019

**Published:** 2018-09-15

**Authors:** Lidewij R. Renaud, Maaike A. Huysmans, Hidde P. van der Ploeg, Erwin M. Speklé, Allard J. van der Beek

**Affiliations:** 1 Department of Public and Occupational Health, Amsterdam Public Health Research Institute, Amsterdam UMC, Vrije Universiteit Amsterdam, Van der Boechorststraat 7, NL-1081 BT Amsterdam, The Netherlands; m.huijsmans@vumc.nl (M.A.H.); hp.vanderploeg@vumc.nl (H.P.v.d.P.); erwin.spekle@arbounie.nl (E.M.S.); a.vanderbeek@vumc.nl (A.J.v.d.B.); 2 Arbo Unie OHS, Diakenhuisweg 25, 2033 AP Haarlem, The Netherlands

**Keywords:** sit-stand workstations, office workers, long-term access, user profiles

## Abstract

*Background*: To decrease the detrimental health effects of prolonged sitting, the implementation of sit-stand workstations is a commonly used intervention for office workers. Most studies on this topic evaluated the effects of newly introduced sit-stand workstations. The objective of this study was to determine how often and how long the standing option is used and how the use of sit-stand workstations is perceived in office workers with long-term access to these workstations. *Methods*: Using an online survey, 1098 office employees responded to questions about frequency of usage of the sit-stand workstation, sitting time, physical activity, and positive and negative perceptions of the use of the sit-stand workstations. *Results*: Based on the frequency of use, three user groups were identified: non-users (32.1%), monthly/weekly users (37.5%) and daily users (30.4%). Non-users reported to sit more, stand less and have longer bouts of sitting, compared to monthly/weekly users, and these differences were even larger compared to daily users. A higher proportion of daily users perceived the use of the sit-stand workstation as being more healthy and appealing and making them more productive and energetic compared to the non-users. A higher proportion of the non-users perceived it as being uncomfortable, distracting, and unpractical, compared to the other user groups. *Conclusions*: The differences between the three identified user groups with respect to sitting, standing and perceptions of sit-stand workstations, might be helpful in tailoring future interventions to reduce occupational sitting time, to increase the reach, effectiveness and sustainability.

## 1. Introduction

Prolonged sitting has been associated with detrimental health outcomes, such as an increased risk for diabetes, heart disease and premature death [1,2]. Especially office workers show high accumulated sitting hours at work [3], indicating that for this group, interventions to reduce sitting time are relevant [4,5,6]. Also, interventions to reduce sitting time at the office, by alternating it with standing or moving, might reduce other health problems, such as musculoskeletal symptoms [7,8]. The most commonly implemented intervention to reduce sitting time at work is the introduction of sit-stand workstations, allowing workers to alternate between sitting and standing, while continuing their work. Recent studies on this topic, mainly focused on the short-term effects (up to 3 months) of the introduction of sit-stand workstations [9,10]. These studies, mostly using objective methods to assess sitting time, showed a reduction in occupational sitting time in intervention groups, compared to the standard office setting. Studies evaluating the short-term effects at 3 month [11] and long-term effects at 12 month [12], showed an attenuation in reduced sitting time, compared to their prior found effects (at 1 and 3 months, respectively). This finding suggests that the number of workers who use the sit-stand workstation decreases over time. Another study found that office workers with long-term access (>6 months) to sit-stand workstations, sit less and stand more compared to employees with access to standard sitting workstations [13]. A recent study on habitual use of the sit-stand workstations, found that participants (*n* = 18), with the majority having access for less than one year, spent 61% of their time at their workstation standing [14]. Most participants in this study requested the workstation for medical reasons or to sit less, so high standing times could be expected. Little is known about actual use of sit-stand workstations and accompanied sitting and standing behaviour in office employees who all have long-term access to these workstations.

Quantitative evaluations of the effect of the use of sit-stand workstations on work performance, have shown either contradicting results [9] or no change compared to sitting [8,15]. For musculoskeletal discomfort, an overall improvement was found for the neck and shoulder region [8] but an exacerbation was found for the leg and foot region [16]. It would be interesting to know what perceptions of potential users on these outcomes are and if these perceptions are reasons for use or non-use of the sit-stand workstations. Qualitative evaluations of the introduction of sit-stand workstations and possible facilitators and barriers for use, have suggested that reasons for office workers to use the standing option were willingness to try something new or the perception of potential health benefits [17]. In other research, facilitating factors to reduce sitting at work were identified as raising awareness or some obligatory strategies [18]. However, major barriers for the use of sit-stand workstations, mentioned in qualitative research, were the perception of decreased work productivity and the persistent habit of sitting [18,19]. McGuckin et al. [20] found that strategies to reduce sedentary behaviour should include multiple strategies to address personal preferences. Whether these and other perceptions of the use of sit-stand workstations were similar for frequent users and non-users of sit-stand workstations, remained unclear in these studies.

The objective of this study was to determine how frequently office workers with long-term access to sit-stand workstation, use the standing option of the workstation. Additionally, we investigated whether different user groups, i.e., non-users, monthly/weekly users and daily users, differed with respect to their self-reported sitting and standing time, physical activity, their positive and negative perceptions of the use of the sit-stand workstations and their perceptions of potential interventions to reduce sitting time at work. Better understanding of the differences in perceptions in different user groups, could contribute to tailored approaches for interventions with a better reach and sustainability to reduce occupational sitting time.

## 2. Materials and Methods

### 2.1. Population

In total 3533 office workers were invited to participate in this cross-sectional survey study. Participants were recruited from a large European semi-governmental organisation, with four different worksites based in three European countries. All worksites had office workers working in similar job types and performing similar computer based work tasks. The entity has been providing all their office employees with sit-stand workstations (several brands and types including ASPA, Maarssen, The Netherlands, all electrical adjustable to standing height) since 1999. Several actions had been taken to inform employees about the correct use of sit-stand workstations (brochures, posters, informational gatherings), but no major additional interventions were implemented in the last year(s). At the company, employees have access to in-company trained colleagues for ergonomic assessments of their workstations and working techniques.

### 2.2. Procedure

Employees were invited by a personal email, providing a link to the online survey, in April 2017. The online survey was built and distributed using the Survalyzer software (Survalyzer BV the Netherlands, Utrecht, The Netherlands). A maximum of two reminders was sent to non-responders, one week and three weeks after the initial invitation. After four weeks, the survey was set offline and the data was locked. The Medical Ethical committee of VU University Medical Center gave approval for this study and assessed it as not subject to the Medical Research (Human Subjects) Act (reference code: 2016.346). Informed consent for participation was obtained in a statement before participants could start the survey.

### 2.3. Survey

The survey contained questions about sedentary behaviour and physical activity inside and outside the office. The use of the sit-stand workstations, positive and negative perceptions of the use of sit-stand workstations and perceptions of potential interventions to reduce sitting time at work, were assessed using a newly developed set of questions, since no validated questions were available on this topic. The content is described in short here below (see Appendix A for specific questions and answering categories). Descriptive characteristics, including gender, age, body weight, body height, educational level, duration of employment, and days working from home, were assessed at the end of the questionnaire. Furthermore, experienced symptoms (pain or discomfort) in eight body parts (wrists and/or hands, elbows, neck and/or shoulders, upper back, lower back, hips and/or thighs, legs and/or knees, and feet and/or ankles) were determined with the modified Nordic questionnaire [21], using a four-item answering scale (never; once in a while; frequently; for a longer period of time). Participants were also asked if they had received any ergonomic instruction regarding their workplace in the past 12 months.

#### 2.3.1. Use of the Sit-Stand Workstation

In Appendix A, the questions on frequency of the use of the sit-stand workstation are specified. Participants could indicate if they used the standing option of the sit-stand workstation at least once per month (yes/no). In case the answer was yes, the frequency of use of the sit-stand workstations was specified using a single-item question with five answering categories: less than once per week but at least once per month; once or twice per week; three to four times per week; once or twice per day; three times or more per day. Also, for users of the workstations, duration per standing episode was assessed, with four answering categories: over 60 min per standing episode; 30–60 min per standing episode; 15–30 min per standing episode; and less than 15 min per standing episode.

Additional questions were asked concerning the actual use of the sit-stand workstation, including “I use the sit-stand workstation at consistent and regular intervals”. Also, they were asked about reasons to switch back to sitting when using the standing option, e.g., “I switch back to sitting because I feel tired” or “…because I switch to a different work task”. All statements (see Appendix A) could be answered on a 5-point Likert scale ranging from strongly agree to strongly disagree. Specific reasons why the standing option could not be used, were assessed for all participants.

#### 2.3.2. Sitting Time and Physical Activity

Sitting time and physical activity were measured using the Workforce Sitting Questionnaire (WSQ), which assessed sitting time during workdays and non-workdays and the Occupational Sitting and Physical Activity Questionnaire (OSPAQ), which assessed sitting, standing and physical activity during work. Both questionnaires showed acceptable validity compared to objectively measured sitting time at work, with rhos varying between 0.35 to 0.48 for the OSPAQ and rhos varying between 0.25 to 0.30 for the WSQ [22]. For standing time at work, the OSPAQ showed acceptable validity in workers having access to a sit-stand workstation, with rho’s up to 0.68 [22]. For moderate-intensity activities and walking at work, a moderate correlation of 0.29 with accelerometer measurements was found [23]. Total time at the desk per working day and longest bouts of sitting were assessed using newly developed questions and answering categories with incremental time scales (see Appendix A). Also, means of transportation (e.g., by car or by bike) to and from work were examined in a one-item question. To examine if participants met the guidelines for physical activity (>30 min MVPA for 5 or more days per week), a single-item question was added with correlations ranging from 0.46 to 0.57 compared to accelerometer data [24].

#### 2.3.3. Positive and Negative Perceptions of the Use of the Sit-Stand Workstation

Statements about positive and negative perceptions of the use of the sit-stand workstations (see Appendix A for the complete overview) were provided with a 5-point Likert answering scale (strongly agree-strongly disagree). Statements on positive perceptions included for example “Using the standing option of my desk at work makes me more productive” or “I believe using the standing option can reduce acute health issues, such as low back pain and musculoskeletal discomfort”. Statements about negative perceptions included “The standing option of my desk is not practical to use” or “I already exercise enough in my leisure time, so standing at work is not necessary”.

#### 2.3.4. Potential Interventions to Reduce Sitting Time at Work

Eight potential interventions (see Appendix A) to reduce sitting time at work, were proposed (e.g., “digital reminders on the computer or phone” or “introducing walking meetings”) with a 5-point Likert answering scale indicating the rate of agreement.

### 2.4. Statistics

Collected data were analysed using SPSS Statistics 22.0 (IBM Inc., Armonk, NY, USA). Frequency of the use of the standing option of the sit-stand workstation was used to identify three different user groups. Body mass index (BMI) was calculated from self-reported body height and weight. Linear outcomes such as BMI, age and sitting time (WSQ) were presented as mean (SD) per user group and group differences were tested using ANOVA with significance level set at *p* < 0.05. Answer categories of the Nordic questionnaire were dichotomised into experiencing symptoms (frequently; for a longer period of time) and not experiencing symptoms (once in a while; never). The single-item physical activity question was dichotomised into meeting the guidelines (5 days or more per week active for 30 min) or not. Differences of ordinal descriptive outcomes between user groups were analysed using a chi square test with *p* < 0.05.

To analyse outcomes on statements concerning positive and negative perceptions of the use of sit-stand workstations or potential interventions to reduce sitting, the 5-point Likert scales, were dichotomised into agree (agree and strongly agree) or not agree (neutral, disagree and strongly disagree). For negative statements, the neutral option was clustered with both agree options, instead of both disagree options. Logistic regression analyses were conducted to analyse outcomes and odds ratios (ORs) were calculated including 95% confidence intervals for the different user groups, with non-users as reference category. Models were inspected on confounding for age, gender and BMI. If outcomes (ORs) did not change >10%, then unadjusted models were presented. In Appendix B (Table A3, Table A5, Table A6 and Table A7) the complete overview of outcomes on the 5-point Likert scales and on work tasks and preferred postures during execution (Table A8 and Table A9) is presented for the different user groups.

## 3. Results

### 3.1. Respondents

**Figure 1:**
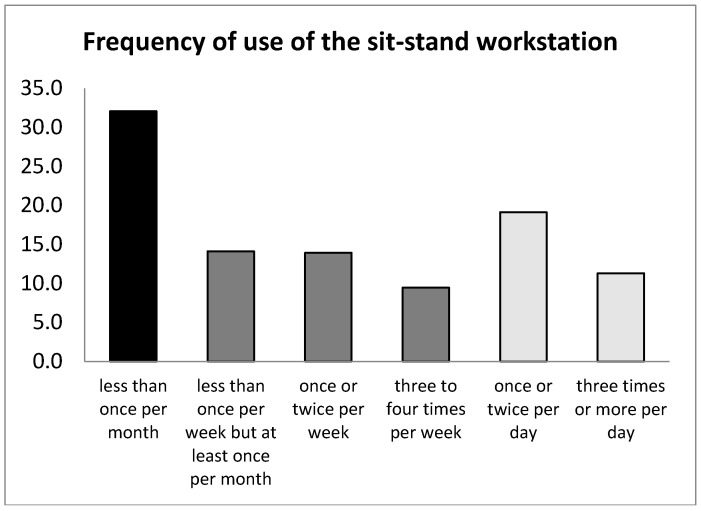
Percentages of self-reported frequency of the use of the sit-stand workstation, with total N = 1098. Black = non-users, dark grey = monthly/weekly users, light grey = daily users.

Of the 3533 invitees, 1424 activated the survey link and 1312 employees consented to take part in the study (37.1%). There were 214 participants providing incomplete data (dropping out before completing the survey), which resulted in a total of 1098 participants with eligible and complete data entries for data analyses. In Figure 1 an overview of frequencies of the use of the sit-stand workstation is shown. Three user groups could be identified, based on these frequencies:

- *Non-users*: 32.1%, using the standing option not at all or less than once per month;
- *Monthly/weekly users*: 37.5%, using the standing option at least once per month up to three to four times per week;
- *Daily users*: 30.4%, using the standing option at least once per day.

### 3.2. Descriptive Characteristics

In Table 1, the descriptive characteristics of the three user groups and the total population are presented. 

Participants were mainly male (64.6%), had a mean age of 46.5 (SD ± 7.8) years and a body mass index (BMI) of 24.6 (SD ± 3.7) kg/m^2^. Overall, the population was highly educated (44.7% MSc and 33.6% PhD degree) and was working fulltime (38.6, SD ± 6.1 h per week on average). The population consisted of many different nationalities, the largest groups being German (25.7%) and French (17.6%). Between user groups significant differences were found for age, duration of employment and BMI, with non-users being older, having a higher duration of employment and a higher BMI. Self-reported musculoskeletal symptoms in different body regions were mostly reported in the neck/shoulder region (34.3%), with a significantly higher percentage reported in the monthly/weekly users (39.6%), opposed to non-users (31.5%) and daily users (30.8%). About half (50.9%) of the employees had received ergonomic instructions regarding their workplace in the past year, with significantly more monthly/weekly users (55.2%) compared to non-users (43.2%).

### 3.3. Use and Non-Use of the Sit-Stand Workstation

The majority of non-users (77.6%) indicated that there was no specific reason for them not to use the sit-stand workstation (Appendix B). In Table 2, an overview of statements about the use of the standing option and reasons to switch back to sitting when standing, is provided for weekly/monthly and daily users. The standing option was mostly used for 15–30 min per episode (44.6%). Still, 13.9% of all users reported to use the standing option for over 60 min per standing episode. Daily users were more likely to use the sit-stand workstations at consistent and regular intervals compared to monthly/weekly users. They also agreed more to use prompts to remember to use the standing option, although this was only 15.0% compared to 7.8% of monthly/weekly users. Reasons to switch back to sitting did not differ between the two user groups. For both groups main reasons were of physical grounds (feeling tired, feeling discomfort, feeling they stood for long enough) or because they switched to another work task.

### 3.4. Sitting Time and Physical Activity

In Table 3 outcomes on sitting and standing time and physical activity are shown for the three user groups and for the total population. Daily users reported the lowest occupational sitting time and highest occupational standing time, compared to monthly/weekly users and non-users. Total sitting time during a workday, measured using the WSQ, was significantly different between the user groups, with daily users, showing lower sitting time (mean = 9.3, SD ± 2.4 h) than monthly/weekly users (mean = 10.6, SD ± 2.0 h) and non-users (mean = 11.1, SD ± 2.1 h). Daily users also reported to sit significantly less than non-users and monthly/weekly users during work and during non-workdays. A similar trend was found for longest uninterrupted time being seated at the desk, with 9.0% of the daily users reporting to sit for 2 h or more, opposed to 21.1% of the monthly/weekly users and 25.6% of the non-users. For transportation to work by car, a similar difference was shown between groups, with 29.6% of daily users, 32.5% of the weekly/monthly users and 44.3% of the non-users reporting the car as their main mode of transportation. Also, meeting the guidelines for being physically active (30 min per day for at least five days per week) showed a similar (not statistically significant) trend, with 34.7% of the daily users meeting this guideline and 29.5% of the non-users.

### 3.5. Positive Perceptions of the Use of Sit-Stand Workstations

In Table 4, an overview of dichotomised positive perceptions of the use of sit-stand workstations is presented for the three different user groups. We found for all perceptions a significantly larger proportion of monthly/weekly users and of daily users agreeing with these statements compared to the non-users (reference group). Especially for perceptions stating that the use of the standing option makes one more productive and is appealing to them, differences in agreement between non-users and daily users were large, which was also reflected in high ORs. Although the OR (=16.57) was highest for the perception that the use of the standing option increases productivity, the percentage of agreement in daily users was still relatively low (32.6%). Overall, agreement with statements about a positive influence of the use of sit-stand workstations on health, were higher in all groups, with 91.0% of daily users agreeing that using the standing option is beneficial for their health opposed to 50.6% in non-users.

### 3.6. Negative Perceptions of the Use of Sit-Stand Workstations

In Table 5, an overview of negative perceptions of the use of sit-stand workstations is shown for the three different user groups. Non-users mostly agreed and daily users mostly disagreed with these statements. Only the statement about forgetting to use the standing option was mostly agreed upon by the weekly/monthly users (88.0%) with an OR (95% CI) of 1.85 (1.23–2.76) compared to non-users. For all other statements, ORs were all <1 for monthly/weekly users and daily users compared to non-users, indicating that non-users were more likely to agree with these negative statements than users. The statement “the standing option is not practical in use” provided the lowest OR (=0.07), with almost half (46.2%) of the non-users showing agreement compared to only 6.0% of daily users. Also high percentages of the non-users agreed with the use of the standing option causing physical discomfort (77.3%) and standing at work is not necessary because enough exercise is done in leisure time (62.5%).

### 3.7. Potential Interventions to Reduce Sitting Time at Work

In Table 6, an overview per user group is provided for the perceived feasibility of potential interventions to reduce sitting time at work. None of the interventions was considered highly feasible, with most scoring well below 50% agreement. A wide variety in what office workers perceived as feasible interventions was found for the different user groups of the sit-stand workstations. Forced interruptions by computer software and support of their colleagues, were the least perceived as potential interventions in all user groups. For all user groups, most agreement was found for digital reminders (significantly higher for weekly/monthly users with 51.6% agreement) and a training about health promotion in the workplace (significantly higher for daily users with 52.0% agreement). For non-users, changes in office environment, scored the highest percentage of agreement (40.9%).

## 4. Discussion

The objective of this study was to determine how often and how long the standing option is used and how the use of sit-stand workstations is perceived in a population of office workers with long-term access to these workstations. In a population of 1098 office workers we identified three user groups, with 32.1% being a non-user, 37.5% being a monthly/weekly user and 30.4% being a daily user of the sit-stand workstation. Between the three user groups, several differences were found with respect to sitting time and perceptions of the use of the sit-stand workstations. These results indicated that during workdays, daily users sat on average 108 min less and monthly/weekly users sat on average 30 min less compared to non-users. Also for standing, differences between the three user groups were found with daily users standing 15% of their occupational time, compared to 5% for the other user groups. In a recent study, similar differences between users and non-users of sit-stand workstations were found for sitting (66 min per day) and standing (60 min per day), although office workers with long-term access and office workers without access to sit-stand workstations were compared [13]. In a review on newly introduced sit-stand workstations, a pooled effect of 100 min reduction in sitting time was found per eight-hour workday on the short term (~3 months) [10], indicating that sit-stand workstations are effective in reducing sitting time on a group level. Still, effects may attenuate over time at the long-term [12] and, as indicated by the current results, access to sit-stand workstations does not guarantee a reduction in sitting time for the individual user. For the three user groups, differences in the use of the sit-stand workstations were accompanied with considerable differences in perceptions of the use of the sit-stand workstations. Insight in these perceptions might contribute to the development of better tailored interventions to reduce occupational sitting time, with increased reach and sustainability.

Not surprisingly, positive perceptions were all more agreed upon by daily users, while negative perceptions were mostly more agreed upon by non-users. A higher proportion of daily users perceived the use of the sit-stand workstation as being more healthy and appealing and making them more productive and energetic compared to the non-users. Whereas a higher proportion of the non-users perceived the use of the sit-stand workstation as being uncomfortable, distracting, and unpractical. Outcomes for weekly/monthly users were found to be in between the other two user groups. Since the cross-sectional nature of the study, unfortunately, no conclusions can be drawn on causality. Therefore, it is unclear whether certain positive or negative perceptions may influence the use of sit-stand workstations or if the frequency of use could influence perceptions of use. In the following paragraphs we provide a more detailed description of the three different user profiles.

### 4.1. Non-User Profile

Non-users seemed to be the most sedentary, with an average total sitting time on workdays of 11.1 (SD ± 2.1) hour, which indicates an increased mortality risk [25]. Non-users seemed in general not very positive about the use of their sit-stand workstation; the majority (77.3%), found that the use of the standing option caused physical discomfort and 46.2% found it not practical in use. Practical disadvantages have been mentioned before as a barrier for use in a qualitative evaluation of a sit-stand workstation intervention [17]. Only half (50.6%) of the non-users perceived the use of the standing option as beneficial for their health. The present study showed that there was no intervention strategy to which non-users convincingly agreed, with a highest agreement of 40.9% for changes in office environment, such as central placement of bins and printers as a potential intervention to reduce sitting time.

### 4.2. Monthly/Weekly User Profile

In the monthly/weekly users, sitting time on workdays appeared to be high (10.6, SD ± 2.0 h). The weekly/monthly users most frequently reported to forget to use the standing option of their workstation (88%) and few monthly/weekly users indicated to use the standing option at consistent and regularly intervals (13.6%). Only 8.0% of them perceived the use of the standing option as making them more productive. In qualitative research on strategies to influence sedentary time at work, the fear for being unproductive has been stated as a general barrier to adopt these strategies [18]. About half (51.6%) of the monthly/weekly users found digital reminders a potential intervention to reduce sitting at work, followed by 43.3% agreeing with a training in health promotion at the workplace.

### 4.3. Daily User Profile

As expected, daily users sat the least during workdays (9.3, SD ± 2.4 h) and stood the most (about 15% of the work week). A majority indicated that using the standing option reduces acute health issues, such as low back pain (83.8%), and the risk of developing chronic diseases (64.0%). The majority of daily users, used the standing option one to two times per day (62.9%), which is similar to a study among active users of sit-stand workstations that reported 1.7 times per day [14]. Although it is unclear how often breaking up sitting is optimal in terms of health [26], it seems that one or two times per working day would not be enough and large bouts of sitting could still be accumulated. Daily users had the highest agreement for a training on health promotion (52.0%), followed by digital reminders (43.9%) as a potential intervention strategy to reduce sitting at work.

### 4.4. Potential Interventions

For all user profiles, there did not seem to be one intervention strategy that stood out. The most preferred intervention strategies to reduce sitting at the workplace were barely supported by half of the participants, which suggests that single component interventions are not likely to be highly successful at a group level. A systematic review has shown that multi-component interventions, combining environmental, organisational and individual components, had a greater effect on the reduction of sitting time than solely introducing sit-stand workstations [27]. Also, specifically targeting a reduction of sitting instead of increasing physical activity has been identified as an important strategy to reduce sitting time [9,28]. Still, it remained unclear if multi-component interventions, aimed at reducing sitting time at work, contribute to the reduction sitting time by increasing the number of individual users of the intervention components, or by decreasing sitting time further in a small group of already active users. To reach all potential users of an intervention, different user groups may require different approaches.

An important insight from the current study is that having access to a sit-stand workstation did not result in actual usage for over one third of the studied office population. Firstly, for this non-user profile, in order to reduce long-term health risks of prolonged sitting, it would be important to increase interruptions of bouts of sitting [26,29]. However, given their negative perceptions of the use of sit-stand workstations, other environmental intervention strategies may be more feasible; such as the central placement of bins and printers, which was most agreed upon in the current study. Practitioners have also indicated this type of obligating environmental changes as a strategy with high potential in an earlier study [19]. Secondly, for the monthly/weekly user profile, there seemed to be a high potential to increase the use of the sit-stand workstations by making it an appealing daily routine. Since they indicated to forget to use the standing option and given their preferences for potential interventions, the emphasis for this user profile could be on providing (digital) reminders. Thirdly, for the daily user profile, it might be beneficial to emphasise on increased alternation between sitting and standing, by means of a health promotion intervention and by using (digital) reminders. For this group the main message could be that any prolonged posture should be avoided [30], since standing too long is also identified as a health risk, for example for developing varicose veins [31].

For practitioners, to tailor multi-component interventions to user profiles, a first step in the development could be to identify the user profiles in the specific target population, and to adjust the intervention components accordingly. Pre-intervention surveys have successfully been used before, to personalise interventions to reduce sitting at work [32].

Further research should focus on the different user groups, assessing reasons for use and non-use of sit-stand workstations in populations with long-term access. To get a profound understanding on why non-users do not use the workstation and in what circumstances they would change their behaviour, as well as a better insight in appropriate intervention strategies, qualitative research might be especially helpful.

### 4.5. Strengths and Limitations

A major strength of this study is the large international office population with long-term access to sit-stand workstations. Still, important limitations should be considered while interpreting the results. The study was cross-sectional, implying that it was not possible to determine causality in the observed associations. Although several validated questionnaires were used, results should be interpreted with caution, as self-reported data is prone to recall and social desirability bias [33]. The profiling of the three user groups was based on a non-validated, single question about frequency of use of the sit-stand workstations. Although this was a very specific item, similar questions, about breaking up occupational sitting, have been used in prior research [34] and have shown fair validity for frequency of breaks in sitting per work hour (rho = 0.39) when tested against accelerometer data [35]. Because of possible selection bias, with users of the sit-stand workstation being more willing to fill out a questionnaire about sit-stand workstations, the group of non-users might be larger in the total office worker population. The purpose of this study was to study associations rather than surveillance, and caution should be taken when interpreting prevalence estimates of the current results. Furthermore, the study population was highly educated, which was representative for the company under study, but caution should be taken when translating these results to lower educated office populations. Still, many desk-based office populations are highly educated [36].

## 5. Conclusions

In a large population of office workers with long-term access to sit-stand workstations, three user group profiles were identified with about one third being non-users, one third being a weekly/monthly users, and one third being daily users. The profiles showed clear dose-response associations for sitting and standing time, and for perceptions of the use of sit-stand workstations, e.g., the more the sit-stand workstation was used, the less sedentary people were and the more positively the workstation was perceived. When implementing a multicomponent intervention to reduce sitting time at work, the identified user profiles might be helpful in tailoring the intervention to increase its reach, effectiveness and sustainability.

## Figures and Tables

**Table 1:** Descriptive characteristics of the three user groups and of the total population.

Outcome	Non-Users	Monthly/Weekly Users	Daily Users	Total
Gender, % (n) of males	64.8 (228)	62.8 (258)	66.7 (222)	64.6 (708)
Age, mean (SD) years *	47.9 (7.6)	46.1 (7.5)	45.6 (8.2)	46.5 (7.8)
BMI, mean (SD) kg·m^2^ *	25.1 (4.0)	24.4 (3.7)	24.2 (3.4)	24.6 (3.7)
Duration of employment, mean (SD) years *	17.2 (8.5)	14.2 (8.6)	13.5 (8.6)	14.9 (8.7)
Average working hours, mean (SD) hours/week	38.1 (7.2)	38.8 (5.5)	38.8 (5.6)	38.6 (6.1)
Worked at home at least 1 day/week, % (N)	24.4 (86)	25.2 (104)	18.6 (62)	23.0 (252)
Educational level, % (N)				
Bachelor	12.8 (45)	11.4 (47)	9.6 (32)	11.3 (124)
Master	45.7 (161)	42.2 (174)	46.7 (156)	44.7 (491)
Ph.D.	32.1 (113)	35.2 (145)	33.2 (111)	33.6 (369)
Other	9.4 (33)	11.2 (46)	10.5 (35)	10.4 (114)
Primary nationality, % (N)				
Austrian	2.6 (9)	3.9 (16)	3.0 (10)	3.2 (35)
Belgian	7.1 (25)	4.4 (18)	3.0 (10)	4.8 (53)
British	10.8 (38)	6.1 (25)	6.3 (21)	7.7 (84)
Dutch	9.4 (33)	7.8 (32)	3.6 (12)	7.0 (77)
French	22.2 (78)	15.6 (64)	15.3 (51)	17.6 (193)
German	19.3 (68)	27.7 (114)	30.0 (100)	25.7 (282)
Greek	4.3 (15)	3.9 (16)	2.7 (9)	3.6 (40)
Italian	6.5 (23)	10.9 (45)	10.5 (35)	9.4 (103)
Spanish	6.3 (22)	4.9 (20)	6.0 (20)	5.7 (62)
Other	11.5 (41)	14.8 (61)	19.6 (65)	15.3 (169)
Experienced symptoms, % (N) in				
wrists and/or hands	15.6 (55)	15.3 (63)	14.1 (47)	15.0 (165)
elbows	7.4 (26)	9.2 (38)	5.1 (17)	7.4 (81)
neck and/or shoulders **	31.5 (111)	39.6 (163)	30.8 (103)	34.3 (377)
upper back	14.5 (51)	18.7 (77)	15.3 (51)	16.3 (179)
lower back	22.4 (79)	28.9 (119)	27.2 (91)	26.3 (289)
hips and/or thighs	6.0 (21)	8.7 (36)	8.1 (27)	7.7 (84)
legs and/or knees	11.1 (39)	12.6 (52)	9.9 (33)	11.3 (124)
feet and/or ankles	9.1 (32)	6.6 (27)	7.2 (24)	7.6 (83)
Received ergonomic instructions regarding the workplace in the past 12 months, ** % (N)	43.2 (152)	55.2 (227)	53.6 (179)	50.9 (558)

* Significant difference between the three user groups; ** Significant difference, chi square. *p* < 0.05.

**Table 2:** Average duration of standing episodes and percentages of agreement with statements about the use of the sit-stand workstation and reasons to switch back to sitting for monthly/weekly users and daily users.

Outcome	Monthly/Weekly Users		Daily Users		Total	
	%	(N)	%	(N)	%	(N)
Average duration of using the standing option of the desk						
less than 15 min per standing episode	13.1	(54)	7.2	(24)	10.5	(78)
15–30 min per standing episode	42.7	(176)	47.0	(157)	44.6	(333)
30–60 min per standing episode	30.3	(125)	31.7	(106)	31.0	(231)
over 60 min per standing episode	13.8	(57)	14.1	(47)	13.9	(104)
	**%**	**(N)**	**%**	**(N)**	**OR (95% CI)**	
I use the sit-stand workstation at consistent and regular intervals	13.6	(56)	75.4	(252)	19.54 (13.41–28.46)	
I use prompts to remember to use the standing option	7.8	(32)	15.0	(50)	2.09 (1.31–3.34)	
If my colleagues use the standing option, I often follow their example	36.4	(150)	28.1	(94)	0.68 (0.50–0.93)	
When I use the standing option it contributes to an active lifestyle	59.0	(243)	75.7	(253)	2.17 (1.58–2.99)	
I Switch back to sitting						
because I feel tired	53.6	(221)	58.7	(196)	1.23 (0.92–1.64)	
because I feel discomfort in my body (e.g., legs, back etc.)	59.7	(246)	56.9	(190)	0.89 (0.66–1.19)	
because I experience high work pressure	28.4	(117)	20.4	(68)	0.65 (0.46–0.91)	
because my colleagues/manager request me to	1.2	(5)	1.8	(6)	1.49 (0.45–4.92)	
because I feel like I stood for long enough	65.3	(269)	70.4	(235)	1.26 (0.93–1.72)	
because I switch to a different work task	61.4	(253)	66.2	(221)	1.23 (0.91–1.66)	

Dichotomisation occurred with the neutral answering option added to the disagree option. Unadjusted logistic regression models were used. Odds Ratios (ORs) are shown including 95% Coincidence intervals (CI).

**Table 3:** OSPAQ, Occupational Sitting and Physical Activity Questionnaire; WSQ, Workforce Sitting Questionnaire. Occupational (bouts of) sitting, means of transportation to work, meeting guidelines for being physical active.

OUTCOME	Non-Users		Monthly/Weekly Users		Daily Users		Total	
OSPAQ, median (25-75 IQR)								
Sitting, percentage *	90	(85–92)	85	(80–90)	70	(60–80)	85	(75–90)
Sitting, hours/week *	36.0	(30.4–40.5)	35.2	(29.6–39.6)	30.0	(22.4–35.2)	34.0	(28–38.8)
Standing, percentage *	5	(1–5)	5	(5–10)	15	(10–30)	5	(5–12)
Standing, hours/week *	1.6	(0.4–2.5)	2.2	(1.6–4.0)	6.5	(4.0–12.6)	2.6	(1.5–5.6)
Walking, percentage *	5	(5–10)	5	(5–10)	10	(5–10)	5	(5–10)
Walking, hours/week *	2.3	(1.6–4.0)	2.2	(1.6–4.0)	3.2	(2.0–5.0)	2.5	(1.8–4.2)
WSQ, mean (SD) hours/day								
Sitting workday **	11.1	(2.1)	10.6	(2.0)	9.3	(2.4)	10.4	(2.3)
Sitting non-Workday ***	7.1	(3.2)	6.9	(3.3)	6.2	(2.7)	6.7	(3.1)
Time based at the desk on a typical work day, % (N)								
4 h or less per day	2.8	(10)	1.2	(5)	2.4	(8)	2.1	(23)
4–6 h per day	17.0	(60)	14.1	(58)	16.8	(56)	15.8	(174)
6–8 h per day	57.1	(201)	62.1	(256)	60.2	(201)	59.9	(658)
8 h or more per day	23.0	(81)	22.6	(93)	20.7	(69)	22.1	(243)
Longest period of uninterrupted time seated at the desk, % (N)								
less than 30 min	5.4	(19)	3.4	(14)	12.0	(40)	6.6	(73)
30–60 min	23.9	(84)	20.9	(86)	38.0	(127)	27.0	(297)
1–1.5 h	25.0	(88)	33.5	(138)	25.7	(86)	28.4	(312)
1.5–2 h	20.2	(71)	21.1	(87)	15.3	(51)	19.0	(209)
2 h or more	25.6	(90)	21.1	(87)	9.0	(30)	18.9	(207)
Means of transportation to go to work, % (N)								
By car	44.3	(156)	32.5	(134)	29.6	(99)	35.4	(389)
By public transportation	26.1	(92)	31.8	(131)	35.6	(119)	31.1	(342)
By bicycle	23.6	(83)	28.6	(118)	29.0	(97)	27.1	(298)
By foot	3.4	(12)	4.6	(19)	3.6	(12)	3.9	(43)
other	2.6	(9)	2.4	(10)	2.1	(7)	2.4	(26)
Being physically active for 30 min on at least 5 days per week, % (N)	29.5	(104)	30.4	(125)	34.7	(116)	31.4	(345)

* significant difference, chi square; ** significant difference between all user groups; *** significant difference between daily users and other two user groups. *p* < 0.05.

**Table 4:** Agreement with perceived positive perceptions of the use of the sit-stand workstation and Odds ratios (95% CI) for three user groups, with non-users as reference category.

OUTCOME	Non-Users (Reference)		Monthly/Weekly Users			Daily Users		
	%	(N)	%	(N)	OR (95% CI)	%	(N)	OR (95% CI)
I believe using the standing option can reduce								
the risk of developing chronic diseases (N = 1083)	43.4	(149)	54.8	(224)	1.58 (1.18–2.11)	64.0	(212)	2.32 (1.70–3.16)
acute health issues, such as low back pain and musculoskeletal discomfort (N = 1085)	54.4	(187)	68.9	(281)	1.86 (1.38–2.50)	83.8	(279)	4.34 (3.03–6.22)
Using the standing option of my desk at work (N = 1098)								
makes me more productive	2.8	(10)	8.0	(33)	2.98 (1.45–6.13)	32.6	(109)	16.57 (8.49–32.35)
makes me feel energetic	11.4	(40)	31.3	(129)	3.56 (2.41–5.25)	55.1	(184)	9.57 (6.46–14.18)
is beneficial for my health	50.6	(178)	76.5	(315)	3.17 (2.33–4.32)	91.0	(304)	9.90 (6.45–15.22)
is appealing to me	9.7	(34)	27.9	(115)	3.62 (2.39–5.48)	62.0	(207)	15.25 (10.05–23.13)

Unadjusted logistic regression models were used. 5-point Likert scale was dichotomised with the neutral option included in the disagreement.

**Table 5:** Agreement with perceived negative perceptions of the use of the sit-stand workstation and Odds ratios (95% CI) for three user groups, with non-users as reference category.

OUTCOME	Non-Users (Reference)		Monthly/Weekly Users			Daily Users		
	%	(N)	%	(N)	OR (95% CI)	%	(N)	OR (95% CI)
I often forget to use the standing option (N = 1052)	79.9	(258)	88.0	(359)	1.85 (1.23–2.76)	46.7	(150)	0.22 (0.16–0.31)
I feel uncomfortable using the standing option in presence of my colleagues (N = 991)	24.5	(75)	6.9	(26)	0.23 (0.14–0.37)	4.2	(13)	0.14 (0.07–0.25)
I get distracted when colleagues use their desk in the standing position (N = 965)	20.5	(62)	7.0	(26)	0.29(0.18–0.48)	3.1	(9)	0.12 (0.06–0.26)
The standing option of my desk is not practical to use (N = 1053)	46.2	(153)	17.9	(72)	0.25 (0.18–0.35)	6.0	(19)	0.07 (0.04–0.12)
I do not have time to use the standing option (N = 1037)	36.9	(118)	23.6	(94)	0.53 (0.38–0.73)	8.8	(28)	0.17 (0.11–0.26)
I already exercise enough in my leisure time, so standing at work is not necessary (N = 1061)	61.5	(209)	40.6	(162)	0.43 (0.32–0.58)	21.1	(68)	0.17 (0.12–0.24)
Using the standing option of my desk at work causes physical discomfort (N = 1098)	77.3	(272)	55.8	(230)	0.37 (0.27–0.51)	35.9	(120)	0.17 (0.12–0.23)

Unadjusted logistic regression models were used. 5-point Likert scale was dichotomised with the neutral option included in the agreement.

**Table 6:** Agreement with feasibility of potential interventions to reduce sitting time at work per user group.

OUTCOME	Non-Users (Reference)		Monthly/Weekly Users		OR (95% CI)	Daily Users		OR (95% CI)
	%	N	%	N		%	N	
Digital reminders on the computer or phone (N = 1083)	40.4	139	51.6	211	1.57 (1.18–2.10)	43.9	145	1.16 (0.85–1.57)
Forced interruptions of sitting through computer software (N = 1077)	26.8	92	24.4	99	0.88 (0.64–1.23)	21.6	71	0.75 (0.53–1.07)
Introducing walking or standing meetings (N = 1075)	38.3	132	38.6	156	1.02 (0.76–1.36)	42.0	137	1.17 (0.86–1.59)
Specific instructions on how, when and why to use the standing option of my desk (N = 1078)	31.8	110	38.6	156	1.35 (1.00–1.83)	35.1	115	1.16 (0.84–1.60)
A training about health promotion in the workplace (N = 1077)	36.4	124	43.3	177	1.34 (0.99–1.79)	52.0	170	1.90 (1.39–2.58)
Support of my colleagues (N = 1074)	19.0	65	22.3	90	1.23 (0.86–1.75)	22.6	74	1.25 (0.86–1.82)
Executives and managers acting like role models promoting standing and walking (N = 1074)	26.1	89	30.4	123	1.24 (0.90–1.71)	32.8	108	1.38 (0.99–1.93)
Changes in the office environment, such as central placement of bins and printers (N = 1071)	40.9	138	34.2	139	0.75 (0.56–1.01)	42.4	139	1.06 (0.78–1.44)

Unadjusted logistic regression models were used. Dichotomisation occurred with the neutral answering option added to the disagree option.

## Appendix A. Newly Developed Set of Questions about Sedentary Behaviour and the Use of Sit-Stand Workstations

**Table A1:** Specific questions and answer categories on sedentary behaviour and use of the sit-stand workstations.

Category	Question	Answering options
Use	At work do you use the standing option of your desk at least once per month?	- yes - no
Frequency	How often do you use the standing option of your desk on average?	- three times or more per day - once or twice per day - three to four times per week - once or twice per week - less than once per week but at least once per month
Reasons for non-use	Is there any reason you cannot use the standing desk for a longer period of time?	- no, there is no specific reason - my desk is broken - I am (temporarily) disabled - I am advised to avoid prolonged standing - I wear uncomfortable shoes - I am pregnant - Other, namely:
Duration (not for non-users)	When using the standing option, for how long are you on average in this position?	- less than 15 min per standing episode - 15–30 min per standing episode - 30–60 min per standing episode - over 60 min per standing episode
Use of the standing option (not for non-users)	I use the sit-stand workstation at consistent and regular intervals	5-point Likert scale ^1^
	I use prompts to remember to use the standing option	5-point Likert scale ^1^
	If my colleagues use the standing option, I often follow their example	5-point Likert scale ^1^
	When I use the standing option it contributes to an active lifestyle	5-point Likert scale ^1^
Reasons to switch back to a seated position after the use of the standing option (not for non-users)	because I feel discomfort in my body (e.g., legs, back etc.)	5-point Likert scale ^1^
	because I experience high work pressure	5-point Likert scale ^1^
	because my colleagues/manager request me to	5-point Likert scale ^1^
	because I feel like I stood for long enough	5-point Likert scale ^1^
	because I switch to a different work task	5-point Likert scale ^1^
Occupational Sedentary behavior	On a typical work day, how long are you based at your desk?	- 4 h or less per day - 4–6 h per day - 6–8 h per day - 8 h or more per day
Occupational bouts of sitting	On a typical workday, what is the longest period of uninterrupted time you stay seated at your desk before standing up (go for a coffee, go to a colleague, etc.)	- less than 30 min - 30–60 min - 1–1.5 h - 1.5–2 h - 2 h or more
Means of transportation	In general, what means of transportation do you use to go to and from your work?	- by car - by public transportation - by bicycle - by foot - other, namely
Positive perception of the use of the standing option of a desk.	I believe using the standing option can reduce the risk of developing chronic diseases such as cardiovascular diseases and diabetes	5-point Likert scale *^,2^
	I believe using the standing option can reduce acute health issues, such as low back pain and musculoskeletal discomfort	5-point Likert scale *^,2^
	Using the standing option of my desk at work...	
	makes me more productive	5-point Likert scale ^1^
	makes me feel energetic	5-point Likert scale ^1^
	is beneficial for my health	5-point Likert scale ^1^
	is appealing to me	5-point Likert scale ^1^
Negative perception of the use of the standing option of a desk.	I often forget to use the standing option	5-point Likert scale *^,2^
	I feel uncomfortable using the standing option in presence of my colleagues	5-point Likert scale *^,2^
	I get distracted when colleagues use their desk in the standing position	5-point Likert scale *^,2^
	The standing option of my desk is not practical to use	5-point Likert scale *^,2^
	I do not have time to use the standing option	5-point Likert scale *^,2^
	I already exercise enough in my leisure time, so standing at work is not necessary	5-point Likert scale *^,2^
	Using the standing option of my desk at work......causes physical discomfort	5-point Likert scale ^1^
Feasible interventions to reduce sedentary behavior at work	Digital reminders on the computer or phone	5-point Likert scale ^1^
	Forced interruptions of sitting through computer software	5-point Likert scale ^1^
	Introducing walking or standing meetings	5-point Likert scale ^1^
	Specific instructions on how, when and why to use the standing option of my desk	5-point Likert scale ^1^
	A training about health promotion in the workplace	5-point Likert scale ^1^
	Support of my colleagues	5-point Likert scale ^1^
	Executives and managers acting like role models promoting standing and walking	5-point Likert scale ^1^
	Changes in the office environment, such as central placement of bins and printers	5-point Likert scale ^1^
Preferred postures, sitting down, standing up or both equally and (in some cases) while walking, during work tasks	Handling email	Posture categories ^3^
	Typing documents	Posture categories ^3^
	Reading documents	Posture categories ^3^
	Searching the internet/intranet	Posture categories ^3^
	Being on the phone	Posture categories ^3^
	One-on-one meetings	Posture categories ^3^
	Having group meetings	Posture categories ^3^
	Having lunch	Posture categories ^3^

Specific answer categories: ^1^ 5 point Likert scale: strongly agree, agree, undecided, disagree, strongly disagree; ^2^ 5 point Likert scale *. strongly agree, agree, undecided, disagree, strongly disagree, not applicable; ^3^ Posture categories: sitting down, standing up, sitting or standing equally, walking, not applicable.

## Appendix B. Additional Results

**Table A2:** Reasons for non-use.

Reasons for Non-Use	Non-Users		Monthly/Weekly Users		Daily Users		Total	
	%	N	%	N	%	N	%	N
Is There a Specific Reason not to Use the Desk? (Option to Tick More than One Box)								
No there is no specific reason	77.6	273	85.4	352	87.7	293	83.6	918
My desk is broken							0.4	4
I am (temporarily) disabled	1.4	5	1.2	5	1.2	4	1.3	14
I am advised to avoid prolonged standing	4.3	15	4.4	18	6.0	20	4.8	53
I wear uncomfortable shoes	1.7	6	3.4	14	2.1	7	2.5	27
I am pregnant							0.4	4
Other	20.5	72	11.9	49	8.4	28	13.6	149

Reasons for non-use of the sit-stand workstations. Answers specified in the ‘other’ open answering option, were categorised as: tiresome/work pressure, discomfort in body, medical reasons, concentration problems, facilities do not allow it, I do not like it, I forget/habit of sitting, different.

**Table A3:** Use of the standing option and reasons to switch back to sitting.

Use of the Standing Option of the Sit-Stand Workstations	Monthly/Weekly Users		Daily Users		Total	
	%	N	%	N	%	N
I use the sit-stand workstation at consistent and regular intervals						
-strongly disagree	21.8	90	2.1	7	13.0	97
-disagree	51.0	210	10.8	36	33.0	246
-undecided	13.6	56	11.7	39	12.7	95
-agree	11.7	48	42.8	143	25.6	191
-strongly agree	1.9	8	32.6	109	15.7	117
I use prompts to remember to use the standing option						
-strongly disagree	49.5	204	38.3	128	44.5	332
-disagree	38.8	160	37.1	124	38.1	284
-undecided	3.9	16	9.6	32	6.4	48
-agree	6.6	27	9.6	32	7.9	59
-strongly agree	1.2	5	5.4	18	3.1	23
If my colleagues use the standing option, I often follow their example						
-strongly disagree	19.2	79	25.7	86	22.1	165
-disagree	25.2	104	24.9	83	25.1	187
-undecided	19.2	79	21.3	71	20.1	150
-agree	35.0	144	23.1	77	29.6	221
-strongly agree	1.5	6	5.1	17	3.1	23
When I use the standing option it contributes to an active lifestyle						
-strongly disagree	4.1	17	2.4	8	3.4	25
-disagree	10.0	41	3.9	13	7.2	54
-undecided	26.9	111	18.0	60	22.9	171
-agree	44.7	184	43.4	145	44.1	329
-strongly agree	14.3	59	32.3	108	22.4	167
**Reasons to switch back to sitting Switch back to sitting**						
because I feel tired						
-strongly disagree	7.8	32	6.6	22	7.2	54
-disagree	21.1	87	17.7	59	19.6	146
-undecided	17.5	72	17.1	57	17.3	129
-agree	43.2	178	45.2	151	44.1	329
-strongly agree	10.4	43	13.5	45	11.8	88
because I feel discomfort in my body (e.g., legs. back etc.)						
-strongly disagree	8.0	33	8.7	29	8.3	62
-disagree	20.6	85	19.5	65	20.1	150
-undecided	11.7	48	15.0	50	13.1	98
-agree	47.1	194	44.0	147	45.7	341
-strongly agree	12.6	52	12.9	43	12.7	95
because I experience high work pressure						
-strongly disagree	21.6	89	24.9	83	23.1	172
-disagree	33.7	139	37.4	125	35.4	264
-undecided	16.3	67	17.4	58	16.8	125
-agree	20.4	84	18.3	61	19.4	145
-strongly agree	8.0	33	2.1	7	5.4	40
because my colleagues/manager request me to						
-strongly disagree	74.0	305	72.8	243	73.5	548
-disagree	22.1	91	22.5	75	22.3	166
-undecided	2.7	11	3.0	10	2.8	21
-agree	1.2	5	1.2	4	1.2	9
-strongly agree	0.0	0	0.6	2	0.3	2
because I feel like I stood for long enough						
-strongly disagree	7.5	31	6.9	23	7.2	54
-disagree	12.4	51	12.0	40	12.2	91
-undecided	14.8	61	10.8	36	13.0	97
-agree	56.1	231	52.4	175	54.4	406
-strongly agree	9.2	38	18.0	60	13.1	98
because I switch to a different work task						
-strongly disagree	6.3	26	6.6	22	6.4	48
-disagree	16.0	66	15.0	50	15.5	116
-undecided	16.3	67	12.3	41	14.5	108
-agree	50.0	206	50.3	168	50.1	374
-strongly agree	11.4	47	15.9	53	13.4	100

**Table A4:** Subdomains of the Workforce Sitting Questionnaire (WSQ).

Subdomains of WSQ	Non-Users		Monthly/Weekly Users		Daily Users		Total	
	Mean	SD	Mean	SD	Mean	SD	Mean	SD
**Workdays**, mean and SD, sitting hours per day								
During transportation	0.85	0.70	0.74	0.65	0.75	0.70	0.78	0.68
During work	7.12	1.22	6.94	1.18	5.65	1.74	6.61	1.52
During tv time	1.21	1.05	1.02	0.98	0.91	0.82	1.05	0..97
During computer time at home	1.04	0.83	0.95	0.74	0.86	0.72	0.95	0.77
During other leisure activities	0.92	0.98	1.00	0.94	1.13	1.07	1.02	0.99
**non-Workdays**, mean and SD, sitting hours per day								
During transportation	0.79	0.83	.78	0.85	0.69	0.77	.075	0.82
During work	0.69	1.94	0.50	1.56	0.47	1.34	0.55	1.63
During tv time	1.78	1.49	1.57	1.31	1.43	1.09	1.59	1.31
During computer time at home	1.63	1.35	1.58	1.29	1.40	1.05	1.54	1.24
During other leisure activities	2.20	2.15	2.44	2.15	2.23	1.80	2.30	2.05

**Table A5:** Agreement with perceived positive perceptions of the use of the sit-stand workstation.

Positive Perceptions of the Use of the Sit-Stand Workstation	Non-Users		Monthly/Weekly Users		Daily Users		Total	
	%	N	%	N	%	N	%	N
I believe using the standing option can reduce the risk of developing chronic diseases such as cardiovascular diseases and diabetes								
-strongly disagree	5.4	19	1.9	8	2.7	9	3.3	36
-disagree	9.1	32	8.3	34	6.6	22	8.0	88
-undecided	40.6	143	34.7	143	26.3	88	34.1	374
-agree	30.4	107	42.7	176	41.9	140	38.5	423
-strongly agree	11.9	42	11.7	48	21.6	72	14.8	162
-not applicable	2.6	9	0.7	3	0.9	3	1.4	15
I believe using the standing option can reduce acute health issues. such as low back pain and musculoskeletal discomfort								
-strongly disagree	5.4	19	1.7	7	1.5	5	2.8	31
-disagree	7.7	27	3.6	15	3.0	10	4.7	52
-undecided	31.5	111	25.5	105	11.7	39	23.2	255
-agree	39.5	139	54.6	225	53.3	178	49.4	542
-strongly agree	13.6	48	13.6	56	30.2	101	18.7	205
-not applicable	2.3	8	1.0	4	0.3	1	1.2	13
**Using the standing option of my desk at work**								
makes me more productive								
-strongly disagree	12.5	44	7.3	30	1.5	5	7.2	79
-disagree	29.3	103	29.6	122	14.1	47	24.8	272
-undecided	55.4	195	55.1	227	51.8	173	54.2	595
-agree	2.3	8	7.5	31	23.1	77	10.6	116
-strongly agree	0.6	2	0.5	2	9.6	32	3.3	36
makes me feel energetic								
-strongly disagree	9.1	32	3.6	15	0.6	2	4.5	49
-disagree	22.4	79	20.4	84	8.1	27	17.3	190
-undecided	57.1	201	44.7	184	36.2	121	46.1	506
-agree	10.2	36	29.9	123	43.4	145	27.7	304
-strongly agree	1.1	4	1.5	6	11.7	39	4.5	49
is beneficial for my health								
-strongly disagree	1.7	6	0.5	2	0.0	0	0.7	8
-disagree	4.8	17	0.2	1	0.6	2	1.8	20
-undecided	42.9	151	22.8	94	8.4	28	24.9	273
-agree	42.9	151	66.3	273	58.7	196	56.5	620
-strongly agree	7.7	27	10.2	42	32.3	108	16.1	177
is appealing to me								
-strongly disagree	13.1	46	1.2	5	1.5	5	5.1	56
-disagree	33.2	117	18.7	77	3.0	10	18.6	204
-undecided	44.0	155	52.2	215	33.5	112	43.9	482
-agree	9.4	33	26.7	110	48.5	162	27.8	305
-strongly agree	0.3	1	1.2	5	13.5	45	4.6	51

**Table A6:** Agreement with perceived negative perceptions of the use of the sit-stand workstation.

Negative Perceptions of the Use of the Sit-Stand Workstation	Non-Users		Monthly/Weekly Users		Daily Users		Total	
	%	N	%	N	%	N	%	N
I often forget to use the standing option								
-strongly disagree	9.9	35	4.4	18	23.1	77	11.8	130
-disagree	8.5	30	7.5	31	28.1	94	14.1	155
-undecided	6.5	23	4.6	19	10.5	35	7.0	77
-agree	24.1	85	57.5	237	29.6	99	38.3	421
-strongly agree	42.6	150	25.0	103	4.8	16	24.5	269
-not applicable	8.2	29	1.0	4	3.9	13	4.2	46
I feel uncomfortable using the standing option in presence of my colleagues								
-strongly disagree	38.9	137	56.1	231	63.8	213	52.9	581
-disagree	26.7	94	29.6	122	24.0	80	27.0	296
-undecided	10.5	37	4.4	18	1.5	5	5.5	60
-agree	6.5	23	1.5	6	1.8	6	3.2	35
-strongly agree	4.3	15	0.5	2	0.6	2	1.7	19
-not applicable	13.1	46	8.0	33	8.4	28	9.7	107
I get distracted when colleagues use their desk in the standing position								
-strongly disagree	44.9	158	54.1	223	62.3	208	53.6	589
-disagree	23.6	83	29.6	122	22.2	74	25.4	279
-undecided	10.5	37	3.4	14	1.8	6	5.2	57
-agree	5.7	20	2.7	11	0.6	2	3.0	33
-strongly agree	1.4	5	0.2	1	0.3	1	0.6	7
-not applicable	13.9	49	10.0	41	12.9	43	12.1	133
The standing option of my desk is not practical to use								
-strongly disagree	22.2	78	43.7	180	66.2	221	43.6	479
-disagree	28.4	100	36.7	151	23.7	79	30.1	330
-undecided	17.9	63	9.5	39	3.3	11	10.3	113
-agree	19.3	68	6.6	27	2.1	7	9.3	102
-strongly agree	6.3	22	1.5	6	0.3	1	2.6	29
-not applicable	6.0	21	2.2	9	4.5	15	4.1	45
I do not have time to use the standing option								
-strongly disagree	24.1	85	39.3	162	61.1	204	41.1	451
-disagree	33.2	117	34.5	142	26.0	87	31.5	346
-undecided	15.9	56	12.4	51	5.4	18	11.4	125
-agree	13.4	47	8.7	36	2.7	9	8.4	92
-strongly agree	4.3	15	1.7	7	0.3	1	2.1	23
-not applicable	9.1	32	3.4	14	4.5	15	5.6	61
I already exercise enough in my leisure time. so standing at work is not necessary								
-strongly disagree	8.8	31	18.7	77	34.7	116	20.4	224
-disagree	28.4	100	38.8	160	41.3	138	36.2	398
-undecided	25.9	91	18.7	77	14.7	49	19.8	217
-agree	23.9	84	15.8	65	4.8	16	15.0	165
-strongly agree	9.7	34	4.9	20	0.9	3	5.2	57
-not applicable	3.4	12	3.2	13	3.6	12	3.4	37
Using the standing option of my desk at work causes physical discomfort								
-strongly disagree	3.4	12	9.7	40	16.5	55	9.7	107
-disagree	19.3	68	34.5	142	47.6	159	33.6	369
-undecided	41.5	146	35.4	146	27.8	93	35.1	385
-agree	25.9	91	18.7	77	6.3	21	17.2	189
-strongly agree	9.9	35	1.7	7	1.8	6	4.4	48

**Table A7:** Agreement with feasibility of potential interventions to reduce sitting time at work per user group.

Possible Interventions to Reduce Sedentary Behaviour at Work	Non-Users		Monthly/Weekly Users		Daily Users		Total	
	%	N	%	N	%	N	%	N
Digital reminders on the computer or phone								
-strongly disagree	20.9	72	13.2	54	13.6	45	15.8	171
-disagree	22.7	78	19.8	81	21.5	71	21.2	230
-undecided	16.0	55	15.4	63	20.9	69	17.3	187
-agree	32.6	112	38.9	159	35.2	116	35.7	387
-strongly agree	7.8	27	12.7	52	8.8	29	10.0	108
Forced interruptions of sitting through computer software								
-strongly disagree	29.7	102	25.7	104	25.5	84	26.9	290
-disagree	27.1	93	33.3	135	35.0	115	31.8	343
-undecided	16.3	56	16.5	67	17.9	59	16.9	182
-agree	20.1	69	18.0	73	18.2	60	18.8	202
-strongly agree	6.7	23	6.4	26	3.3	11	5.6	60
Introducing walking or standing meetings								
-strongly disagree	15.9	55	10.6	43	10.4	34	12.3	132
-disagree	21.2	73	27.2	110	24.8	81	24.6	264
-undecided	24.6	85	23.5	95	22.7	74	23.6	254
-agree	29.9	103	30.9	125	31.9	104	30.9	332
-strongly agree	8.4	29	7.7	31	10.1	33	8.7	93
Specific instructions on how, when and why to use the standing option of my desk								
-strongly disagree	15.3	53	9.4	38	13.4	44	12.5	135
-disagree	26.9	93	25.7	104	25.3	83	26.0	280
-undecided	26.0	90	26.2	106	26.2	86	26.2	282
-agree	28.6	99	33.9	137	28.0	92	30.4	328
-strongly agree	3.2	11	4.7	19	7.0	23	4.9	53
A training about health promotion in the workplace								
-strongly disagree	13.8	47	9.3	38	8.6	28	10.5	113
-disagree	21.4	73	24.0	98	18.0	59	21.4	230
-undecided	28.4	97	23.5	96	21.4	70	24.4	263
-agree	30.2	103	36.7	150	43.1	141	36.6	394
-strongly agree	6.2	21	6.6	27	8.9	29	7.1	77
Support of my colleagues								
-strongly disagree	16.9	58	12.9	52	12.2	40	14.0	150
-disagree	27.7	95	31.9	129	27.2	89	29.1	313
-undecided	36.4	125	32.9	133	37.9	124	35.6	382
-agree	16.3	56	20.3	82	19.0	62	18.6	200
-strongly agree	2.6	9	2.0	8	3.7	12	2.7	29
Executives and managers acting like role models promoting standing and walking								
-strongly disagree	19.9	68	14.4	58	14.3	47	16.1	173
-disagree	26.4	90	30.9	125	25.2	83	27.7	298
-undecided	27.6	94	24.3	98	27.7	91	26.4	283
-agree	19.9	68	25.5	103	24.3	80	23.4	251
-strongly agree	6.2	21	5.0	20	8.5	28	6.4	69
Changes in the office environment. such as central placement of bins and printers								
-strongly disagree	11.3	38	12.1	49	10.1	33	11.2	120
-disagree	19.0	64	22.9	93	21.6	71	21.3	228
-undecided	28.8	97	30.8	125	25.9	85	28.7	307
-agree	33.2	112	29.3	119	32.6	107	31.6	338
-strongly agree	7.7	26	4.9	20	9.8	32	7.3	78

**Table A8:** Preferred Postures for Work Task Execution.

Preference for Work Task Execution	Non-Users		Monthly/Weekly Users		Daily Users		Total	
Handling email *	%	N	%	N	%	N	%	N
-sitting down	82.1	289	46.9	192	26.7	89	52.1	570
-standing up	1.7	6	3.2	13	12.9	43	5.7	62
-sitting or standing equally	13.4	47	48.4	198	60.1	200	40.7	445
-walking	0.0	0	0.5	2	0.3	1	0.3	3
-not applicable	2.8	10	1.0	4	0.0	0	1.3	14
Typing documents								
-sitting down	93.4	326	78.3	322	53.0	177	75.4	825
-standing up	0.6	2	1.2	5	9.3	31	3.5	38
-sitting or standing equally	3.4	12	20.0	82	37.4	125	20.0	219
-walking	0.0	0	0.0	0	0.3	1	0.1	1
-not applicable	2.6	9	0.5	2	0.0	0	1.0	11
Reading documents								
-sitting down	74.8	261	37.8	154	22.8	76	45.1	491
-standing up	2.6	9	12.0	49	21.6	72	11.9	130
-sitting or standing equally	19.5	68	48.9	199	54.1	180	41.0	447
-walking	0.6	2	1.0	4	1.5	5	1.0	11
-not applicable	2.6	9	0.2	1	0.0	0	0.9	10
Searching the internet/intranet								
-sitting down	76.4	269	31.3	127	19.6	65	42.3	461
-standing up	2.3	8	10.3	42	18.7	62	10.3	112
-sitting or standing equally	18.5	65	57.4	233	60.1	199	45.6	497
-walking	0.0	0	0.2	1	0.6	2	0.3	3
-not applicable	2.8	10	0.7	3	0.9	3	1.5	16
Being on the phone								
-sitting down	57.2	199	38.4	156	24.1	80	40.1	435
-standing up	8.6	30	11.3	46	22.0	73	13.7	149
-sitting or standing equally	26.4	92	41.6	169	47.6	158	38.6	419
-walking	4.6	16	7.6	31	5.1	17	5.9	64
-not applicable	3.2	11	1.0	4	1.2	4	1.7	19
One-on-one meetings								
-sitting down	60.5	211	49.5	202	43.3	143	51.1	556
-standing up	10.9	38	12.7	52	17.6	58	13.6	148
-sitting or standing equally	23.5	82	32.8	134	32.1	106	29.6	322
-walking	1.1	4	1.2	5	1.8	6	1.4	15
-not applicable	4.0	14	3.7	15	5.2	17	4.2	46
Having group meetings								
-sitting down	79.8	281	77.8	318	73.3	242	77.1	841
-standing up	3.1	11	2.7	11	7.0	23	4.1	45
-sitting or standing equally	12.2	43	14.4	59	12.7	42	13.2	144
-walking	0.3	1	0.0	0	0.9	3	0.4	4
-not applicable	4.5	16	5.1	21	6.1	20	5.2	57
Having lunch								
-sitting down	93.1	325	92.2	378	91.6	305	92.3	1008
-standing up	0.0	0	0.0	0	1.2	4	0.4	4
-sitting or standing equally	2.3	8	4.1	17	3.3	11	3.3	36
-walking	0.9	3	0.7	3	1.2	4	0.9	10
-not applicable	3.7	13	2.9	12	2.7	9	3.1	34

**Table A9:** Percentages of Work Task Content.

	Median (25–75 IQR)	Median (25–75 IQR)	Median (25–75 IQR)	Median (25–75 IQR)
Work Task Content				
Handling email	5.0 (5.0–20)	5.0 (4–15)	5.0 (5–15)	5.0 (5–15)
Typing documents	20.0 (10–25)	20.0 (10–25)	20.0 (10–25)	20.0 (10–25)
Reading documents	30.0 (15–40)	30.0 (20–42)	30.0 (20–42)	30.0 (20–40)
Searching intranet/internet	10.0 (5–25)	10.0 (5–25)	10.0 (5–25)	10.0 (5–25)
On the phone	2.5 (1–5)	2.0 (1–5)	3.0 (1–5)	2.22 (1–5)
In meetings	5.0 (1–10)	4.0 (1–6)	4.7 (1–10)	4.59 (1–10)
Print, scan or copy	2.5 (1–5)	3.0 (1–5)	4.0 (2–5)	3.0 (1–5)
Other (coffee/lunch breaks)	9.0 (5–10)	9.8 (5–11)	10.0 (5–10)	10.0 (5–10)
